# Soil microbial trait-based strategies drive metabolic efficiency along an altitude gradient

**DOI:** 10.1038/s43705-021-00076-2

**Published:** 2021-12-03

**Authors:** Jiao Feng, Xiao-Min Zeng, Qianggong Zhang, Xin-Quan Zhou, Yu-Rong Liu, Qiaoyun Huang

**Affiliations:** 1 State Key Laboratory of Agricultural Microbiology, Huazhong Agricultural University, Wuhan, 430070 China; 2 College of Resources and Environment, Huazhong Agricultural University, Wuhan, 430070 China; 3 Key Laboratory of Tibetan Environment Changes and Land Surface Processes, Institute of Tibetan Plateau Research, Chinese Academy of Sciences, Beijing, 100101 China

**Keywords:** Microbial ecology, Biogeochemistry

## Abstract

Trait-based approaches provide a candidate framework for linking soil microbial community to ecosystem processes, yet how the trade-offs in different microbial traits regulate the community-level metabolic efficiency remains unknown. Herein we assessed the roles of the microbial taxa with particular trait strategies in mediating soil microbial metabolic efficiency along an altitude gradient on the Tibetan Plateau. Results showed that soil microbial metabolic efficiency declined with increasing altitude, as indicated by the increasing metabolic quotient (microbial respiration per unit biomass, qCO_2_) and decreasing carbon use efficiency (CUE). Both qCO_2_ and CUE were predominantly predicted by microbial physiological and taxonomic attributes after considering key environmental factors including soil pH, substrate quantity and quality. Specifically, the reduced metabolic efficiency was associated with higher investment into nutrient (particularly for phosphorus) acquisitions via enzymes. Furthermore, we identified key microbial assemblies selected by harsh environments (low substrate quality and temperature) as important predictors of metabolic efficiency. These results suggest that particular microbial assemblies adapted to nutrient limited and cold habitats, but at the expense of lower metabolic efficient at higher altitude. Our findings provide a candidate mechanism underlying community-level metabolic efficiency, which has important implications for microbial-mediated processes such as carbon dynamics under global climate changes.

## Introduction

Soil microorganisms are critical drivers of the global carbon (C) cycle because of their roles in both soil organic C (SOC) decomposition and formation, regulating major C flux between soil and atmosphere [1, 2]. Soil C stock is determined by the balance between microbial organic matter decomposition and biomass build-up [3]. Therefore, the metabolic efficiency of microbial community such as metabolic quotient (microbial respiration per unit biomass, qCO_2_) and C use efficiency (CUE) are fundamental for the rates of ecosystem C storage [3–5]. Recent studies suggest that the inclusion of metabolic efficiency into existing models improves the prediction of soil C cycling under global changes [6, 7]. The metabolic efficiency of microbial communities can vary with environmental conditions, and are known to be influenced by abiotic factors (primarily climates and substrate quality) [4, 8]. Moreover, previous studies indicated that altered environmental conditions also induced changes in microbial community structure and physiological attributes [9, 10], which may also contribute to changes in community-level metabolic efficiency [11]. However, despite the acknowledgment that intrinsic properties of microbial community are critical to ecosystem functions [12, 13], how these multiple physiological traits inherent to complex microbial community regulate their energy efficiency remains unclear.

A recent trait-based conceptual framework suggests that the trade-offs between multiple physiological traits play fundamental roles in governing soil C cycling under environmental changes [3]. Microbial community is assumed to be inefficient in soils with low substrate quality (i.e., higher soil C:N and C:P ratio) [8, 14], as physiological traits related to resource acquisition (e.g., enzyme secretion) are energetically expensive [15]. Moreover, harsh environments like extreme low temperature and drought could also reduce microbial growth rate and enhance the C cost of maintenance [11, 16]. The trade-offs between different traits could potentially influence the partition of detrital C into biomass production vs. maintenance respiration, causing changes in metabolic efficiency of microbial community as a whole, with important implications for C cycling under global climate changes.

Given that different microbial taxa have distinct physiological trait combinations, community-level physiological changes may be a consequence of shifts in the relative abundance of specific taxa [11, 17]. Interpreting microbial taxonomic composition under the concept of trait-based ecology may hint at a way to further clarify the underlying mechanisms for regulating community-level metabolic quotient in the context of next-generation sequencing and big data. This topic was moved forward by grouping microbial taxa into different life-history strategies according to their growth vs. adaptation strategies, such as *r*- (fast-growing opportunistic species) and *K*-strategists (slow-growing equilibrium species) [18] and the further defined Yield (growth yield)-Acquisition (resource acquisition)-Stress (stress tolerance) strategies [3]. For instance, *β-Proteobacteria* and *Bacteroidetes* are commonly classified as *r*- strategists, while *Acidobacteria* are *K*-strategists [18, 19]. However, the classification based on high phylogenetic levels (such as at the order or phylum levels) should be used with caution, because microbial taxa with similar functions are commonly phylogenetically diverse and taxonomically associated strains may also have different physiological traits [20, 21]. Microbial community could also be grouped into different functional assemblies based on the co-occurrence or association patterns from networks, offering new insights into complex microbial community structure and soil functioning [10, 22]. Yet, we know little about the effects of shifts in microbial assemblies with different functional traits on the metabolic efficiency of microbial community.

Altitude gradient provides a “natural” setting to test the effects of environmental changes on ecosystem processes, because of the drastic changes in climate, biotic and abiotic attributes over short distances within a landscape. Previous studies have reported that low temperature at higher altitude commonly leads to accumulation of soil organic matter with low substrate quality (i.e., higher soil C:N and C:P ratios) [23]. Moreover, soil microbial community composition shifted significantly along altitude gradients [9]. We assumed that (1) the metabolic efficiency of microbial community would be low at higher altitude due to the likely higher energy investments into resource acquisition; (2) the shifts in microbial assemblies with distinct trait strategies may contribute to changes in community-level metabolic efficiency. To test our hypotheses, we collected soil samples from 28 sites along an altitude gradient on the Tibetan Plateau. This region is the highest and largest plateau and has been declared as the “Third Pole” due to its harsh alpine environments [24], providing ideal platform for exploring the trade-offs between different physiological trait-based strategies. We investigated the associations between microbial metabolic efficiency (including qCO_2_ and CUE) and resource acquisition strategies (soil enzymes and ecoenzymatic stoichiometric ratios). Furthermore, we conducted network analysis to explore key microbial functional assemblies that affect the community-level physiological traits and metabolic efficiency.

## Materials and methods

### Site and sampling

This study was conducted along an altitude gradient (ranging from 2974 to 3558 m) at Mount Segrila (29° 34′–29° 37′ N, 94° 19′–94° 22′ E) on the southeastern part of the Tibetan Plateau (Fig. S1). The mean annual temperature (MAT) declines significantly (*R*^2^ = 0.989) from approximately 8.6 to −0.7 °C as the altitude increased, with a decreasing rate of temperature at 0.54–0.73 °C per 100 m [25, 26]. The mean annual precipitation (MAP) ranges between 680 and 1134 mm, with over 80% of the precipitation occurring during the growing season (between May to September) [25]. The MAT and MAP at the elevation above 3100 m are less than 4.2 °C and more than 1000 mm, respectively, indicating that temperature instead of water availability is the dominant growth-limiting factor for biology [27, 28]. Both microbial biomass and the associated functions were maximum during the growing season [29]. The surface soils begin to freeze at the end of October and start to thaw at the beginning of the next March [25]. The dominant vegetation types shift from temperate coniferous and broadleaved mixed forests to frigid dark coniferous forests as the altitude increased [9]. The predominant soil type is Luvisols and Cambisols according to World Reference Base for Soil Resources [30].

Twenty-eight representative sites were selected in August 2018, covering the dominant vegetation types along the altitude gradient. At each site, five 1 m × 1 m sub-plots were located at each corner and the center of a 50 m × 50 m area. Five replicate soil samples (0–10 cm) were collected from the understory or adjacent open grasslands in each site. After removing visible stones, roots and plant materials, collected soil samples were homogenized. Soil samples were kept on ice when transporting to the laboratory and then divided into two sub-samples. One subsample was stored at −20 °C for the analyses of microbial community (i.e., DNA extraction and MiSeq Illumina sequencing). The other subsample was stored at 4 °C for the analyses of biological activities such as enzyme activities and microbial metabolic efficiency.

### Measurement of soil chemical properties

Soil pH was measured with a glass electrode with a 1:2.5 soil/distilled water ratio. SOC was determined by K_2_CrO_7_ oxidation titration method [31]. Soil total N (TN) was quantified by an elemental analyzer (Vario PYRO Cube, Elementar, Germany). Soil total P (TP) was measured using a digestion method [32]. Soil dissolved organic C and N were extracted with deionized water at a ratio of 1:4 (w/v) and determined using a TOC analyzer (vario TOC, Elementar, Germany). Soil NH_4_^+^ and NO_3_^−^ were determined by a colorimetric method using a continuous flow analyzer (AA III, BRAN + LUEBBE GmbH, Germany) after being extracted with 2 M KCl. Soil available P was measured by the Olsen bicarbonate method [33].

### Analysis of soil bacterial and fungal communities

Soil DNA was extracted using the MoBio PowerSoil DNA Isolation Kit (MoBio Laboratories, Carlsbad, CA, USA) according to the manufacturer’s instructions. The diversity and composition of bacterial and fungal communities were measured by amplifying the V3–V4 region of the 16S rRNA gene with primer pairs 338F/806R [34], and the ITS gene with primers ITS1F/ITS2R [35], respectively. The purified amplicons were mixed equimolarly, and 2 × 300 bp paired-end sequencing was conducted using an Illumina Miseq sequencer (Illumina Inc., San Diego, USA). We used the UPARSE approach to cluster Operational Taxonomic Units (OTUs or phylotypes) with a picking strategy at 97% sequence similarity. The taxonomic information for bacterial and fungal OTUs were assigned using the SILVA and Unite ribosomal RNA gene database, respectively [36]. The α-diversity (i.e., Shannon index) and community composition for bacteria and fungi were calculated based on 97% OTU similarity of obtained sequences. Amplicon sequencing approach has been widely applied to characterize microbial community composition, although some identified taxa may be inactive or dormant [34, 35].

### Analysis of the putative microbial physiological traits

Physiological traits involved in microbial resource acquisitions were assessed by potential extracellular enzyme assays [3]. All enzyme measurements were conducted using sieved soil (<2 mm) at field moisture within one week after sampling. Soil extracellular enzyme activities related to C (α-1,4-glucosidase [AG], β-1,4-glucosidase [BG], cellobiohydrolase [CBH] and xylanase [XYL]), N (N-acetyl-β-D-glucosaminidase [NAG] and leucine aminopeptidase [LAP]) and P (acid phosphatase [AP]) acquisitions were measured by fluorimetric methods [37]. Briefly, 0.50 g of fresh soil was mixed with 50 mL of deionized water and stirred vigorously using a magnetic stir plate for 15 min. Then, 50 μL of soil homogenate, 100 μL of fluorometric substrate solution (200 μmol L^−1^) and 50 μL of acetate buffer (0.2 mol L^−1^, pH 5.5) were mixed and incubated at 30 °C for 3 h [37]. The released fluorescence was measured using a multifunctional microplate reader (Tecan Spark™ 10 M, Männedorf, Switzerland) at 360 nm excitation and 450 nm emission wavelengths. Extracellular enzyme activities were expressed as nmol h^−1^ g^−1^ soil.

Additionally, specific enzyme activities were calculated by normalizing activities to units per mg MBC, in order to avoid the variations induced by biomass change. Ecoenzymatic vector length (Vlength, relative C: nutrient-acquiring ratio) and angle (Vangle, relative P: N-acquiring ratio) created by the plot of proportional enzymatic C:N and C:P ratios were further calculated to illustrate the relative microbial resource acquisition strategies [38]. Higher Vlength indicates relatively higher C vs. nutrient acquisition strategies, and higher Vangle suggests higher P vs. N acquisition efforts. Soil Vlength and Vangle were calculated using Eqs. (1) and (2), respectively:

1 $${{{{{{{\mathrm{Vlength}}}}}}}}=\sqrt {{x^2} + {y^2}}$$

2 $${{{{{{{\mathrm{Vangle}}}}}}}}\;{{{{{{{\mathrm{(degree) = degrees}}}}}}}}\;{{{{{{{\mathrm{(atan2(x,y))}}}}}}}}$$

where atan2 represents the four-quadrant inverse tangent, *x* and *y* represent the relative C:P and C:N acquiring enzyme ratios, respectively.

### Evaluations of metabolic efficiency

Microbial metabolic efficiency was evaluated using both qCO_2_ and CUE, in which lower qCO_2_ and higher CUE indicate higher metabolic efficiency [5, 39]. Microbial respiration was estimated by an incubation method [40, 41]. The soils were incubated aerobically in 250 mL incubation bottles at 20 °C for 14 days. We chose 20 °C for incubation according to the averaged ground temperature in the growing season along the altitude gradient [25]. The short-term (14 days) aerobic incubations were selected to minimize the effects of changes in soil labile substrate and microbial community on soil respiration [42]. The bottles were sealed using parafilms with small holes that enable the exchange of gas, but minimized evaporation and soil water loss [40]. During incubation, 60% of water holding capacity was maintained by weighting the incubation bottles regularly and adding distilled water to compensate for water loss. The bottles were hermetically closed during the 2 h sampling period, and the concentrations of respired CO_2_ were measured using a gas chromatography (Agilent 7890A, Agilent Technologies, USA). The rate of soil respiration was quantified as mg CO_2_–C g^−1^ dry soil h^−1^. Soil microbial biomass C (MBC) was determined by a chloroform fumigation-extraction method [43]. Soil qCO_2_ was expressed as μg CO_2_–C μg^−1^ MBC h^−1^ [39].

Microbial CUE was calculated indirectly using the biogeochemical-equilibrium model (Eqs. (3–5)) [44, 45]:

3 $${{{{{{{\mathrm{CUE = CUE}}}}}}}}_{{{{{{{{\mathrm{max}}}}}}}}} \times \sqrt {\frac{{{{{S}}}_{{{{{{{{\mathrm{C:N}}}}}}}}} \times {{{S}}}_{{{{{{{{\mathrm{C:P}}}}}}}}}}}{{\left( {{{{K}}}_{{{{C:N}}}}+{{{{S}}}}_{{{{{{{{\mathrm{C:N}}}}}}}}}} \right) \times \left( {{{{K}}}_{{{{{{{{\mathrm{C:P}}}}}}}}}+{{{{S}}}}_{{{{{{{{\mathrm{C:P}}}}}}}}}} \right)}}}$$

4 $${{{S}}}_{{{{{{{{\mathrm{C:N}}}}}}}}} = {{{B}}}_{{{{{{{{\mathrm{C:N}}}}}}}}}/{{{L}}}_{{{{{{{{\mathrm{C:N}}}}}}}}} \times 1/{{{{{{{\mathrm{EEA}}}}}}}}_{{{{{{{{\mathrm{C:N}}}}}}}}}$$

5 $${{{S}}}_{{{{{{{{\mathrm{C:P}}}}}}}}}={{{{B}}}}_{{{{{{{{\mathrm{C:P}}}}}}}}}{{{{/L}}}}_{{{{{{{{\mathrm{C:P}}}}}}}}} \times {{{{{{{\mathrm{1/EEA}}}}}}}}_{{{{{{{{\mathrm{C:P}}}}}}}}}$$

In these functions, EEA_C:N_ was calculated as ln (BG)/ln(NAG + LAP), and EEA_C:P_ was calculated as ln (BG)/ln(AP). Molar C:X ratios of labile substrates were used to estimate *L*_C:N_ and *L*_C:P_. Labile C, N and P were DOC, inorganic N (the sum of NH_4_^+^ and NO_3_^−^) and available P, respectively. B_C:N_ and B_C:P_ were the molar ratios of microbial biomass C:N and C:P, respectively. CUE_max_ was fixed to 0.60 and represented maximum microbial growth efficiency according to thermodynamic constraints based on the saturating Michaelis-Menten formulation. *K*_C:X_ was the half-saturation constant (0.50).

### Statistical analysis

We first identified the patterns of qCO_2_, CUE and microbial resource acquisition strategies (including specific enzyme activities, Vlength and Vangle) along the altitude gradient. Principal coordinate analysis (PCoA) was conducted to evaluate variations in the taxonomic composition of bacterial and fungal community. Statistical differences in the microbial community composition were tested using the permutational multivariate analysis of variance (PERMANOVA) by “vegan” package in R 4.0.2 (http://cran.r-project.org/). We conducted a Random Forest analysis to identify the statistically significant predictors of qCO_2_ and CUE using the “rfPermute” package [46]. We compared the percentage increases in the mean squared error (i.e., %IncMSE) to evaluate the relative importance of different variables in predicting qCO_2_ and CUE.

We further conducted a co-occurrence network and identified main ecological clusters (modules or assemblies) of strongly correlated OTUs. To reduce rare OTUs, we removed OTUs with relative abundances less than 0.01% of bacterial and fungal sequences, respectively [22]. The co-occurrence network was inferred according to the Spearman correlation matrix calculated using the “WGCNA” package. The nodes in networks represent OTUs and the edges connecting different nodes represent correlations between OTUs. To reduce false positive results, we adjusted all *P*-values for multiple correlations using Beniamini and Hochberg false discovery rate (FDR) [47]. Robust correlations with the Spearman correlation coefficients > 0.60 and FDR adjusted *P*-values < 0.01 were selected to construct the co-occurrence networks. The network was visualized with Gephi (http://gephi.github.io/). The relative abundance of each module was calculated by averaging the standard relative abundances (z-score) of all taxa belonged to each module [48].

To identify essential microbial assemblies affecting qCO_2_ and microbial physiological traits, we further conducted a Random Forest analysis by incorporating all bacterial and fungal modules into the model. The relationships between the relative abundances of key microbial modules and environmental factors and microbial physiological traits were evaluated using Spearman’s rank correlation analysis. Similarly, we used Spearman’s rank correlation analysis to evaluate the relationships of different genus in key microbial modules with qCO_2_, CUE and microbial physiological traits, in order to identify major taxa driving the linkages between qCO_2_ and different physiological traits.

## Results

### Microbial metabolic efficiency and resource acquisition traits along the altitude gradient

**Fig. 1:**
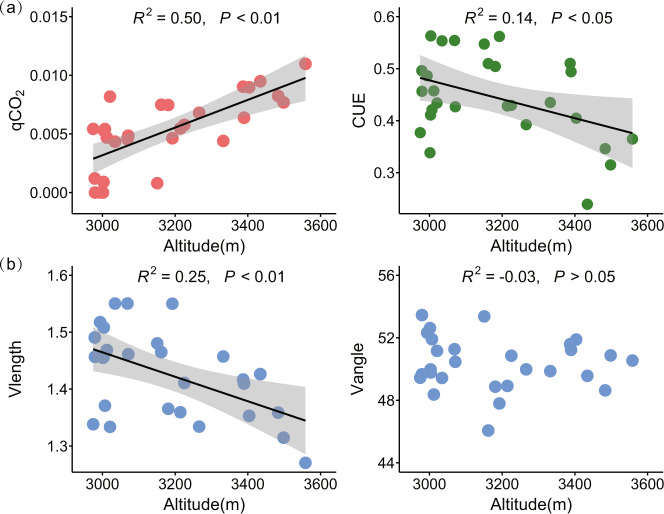
Patterns of microbial metabolic efficiency and resource acquisition strategies along the altitude gradient. **a** Metabolic efficiency, including metabolic quotient (qCO_2_, respiration per unit biomass, μg CO_2_–C μg^−1^ MBC h^−1^) and carbon use efficiency (CUE); **b** microbial resource acquisition strategy. The Vlength quantified the relative C vs. nutrient (N and P)-acquiring enzyme activities, and lower Vlength indicated relative higher nutrient acquisition strategies (relative to C); the Vangle quantified the relative P vs. N-acquiring enzyme activities, and higher Vangle represented relatively higher P vs. N acquisition strategies.

**Fig. 2:**
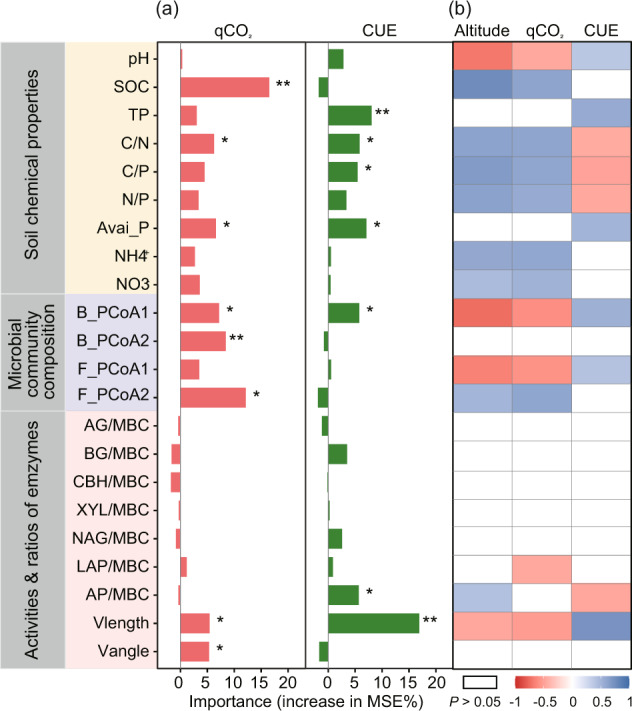
Major predictors of soil metabolic efficiency along the altitude gradient. **a** The percentage increases in the mean squared error (%IncMSE) based on Random Forest analysis; **b** relationships of metabolic quotient (qCO_2_) and carbon use efficiency (CUE) with selected biotic and abiotic attributes. SOC, soil organic C; TP, total P; Avai_P, available P; B_PCoA1, B_ PCoA2, F_PCoA1 and F_PCoA2 were the two dimensional PCoA ordination of taxonomic composition for bacterial and fungal community, respectively. AG, α-1,4-glucosidase activities; BG, β-1,4-glucosidase; CBH, cellobiohydrolase; XYL xylanase; NAG, N-acetyl-β-D-glucosaminidase; LAP, leucine aminopeptidase; AP, acid phosphatase. Enzyme/MBC represents specific enzyme activities calculated by normalizing activities to units/mg MBC. *P < 0.05; **P < 0.01.

Microbial metabolic quotient (qCO_2_) at a community level increased (*P* < 0.01), while CUE decreased with increasing altitude (*P* < 0.05, Fig. 1a). SOC, soil NH_4_^+^ and NO_3_^−^ increased significantly with increasing altitude, while there were no significant changes in soil TP and available P as the altitude increased. Soil C:N:P stoichiometric ratios shifted significantly, with soil C/N, C/P and N/P ratios all increased with increasing altitude (Fig. 2b). Likewise, there were significant shifts of microbial resource acquisition strategies toward higher investments in nutrients compared to C (i.e., declining Vlength) with increasing altitude (Fig. 1b). In particular, specific AP activities that standardized by per unit MBC enhanced significantly as the altitude increased (Fig. 2b). The relative P vs. N-acquiring enzyme activities (i.e., Vangle) was generally higher than 45°, but did not varied with increasing altitude (Fig. 1b). The specific enzyme activities associated with C (including AG, BG, CBH and XYL) and N (including NAG and LAP) acquisitions showed no significant variations along the altitude gradient (Fig. 2b).

### Linking biotic and abiotic factors to metabolic efficiency

Both Random Forest and correlation analysis consistently showed significant associations of microbial taxonomic and physiological traits with metabolic efficiency (Fig. 2). Shifts in microbial resource acquisition strategies (including Vlength and Vangle) and microbial community composition were essential predictors of qCO_2_, after considering essential abiotic attributes including soil pH, substrate quantity and quality (Fig. 2a). Regression analysis further indicated that qCO_2_ correlated negatively with Vlength and specific N-acquiring enzyme activities (Fig. 2b). Likewise, CUE could also be predicted by microbial taxonomic and physiological attributes, including the first dimensional PCoA ordination of bacterial community (B_PCoA1), Vlength and specific P-acquiring enzyme activities (Fig. 2). Soil CUE showed positive relationships with Vlength, but negative associations with specific P-acquiring enzyme activities. In addition, soil abiotic factors such as SOC, TP, C/N, C/P and available P were also significant predictors of variations in metabolic efficiency. Soil qCO_2_ was positively related to SOC, soil NH_4_^+^, NO_3_^−^ concentrations and soil C/N, C/P and N/P ratios. In contrast, CUE showed overall negative associations with soil stoichiometric ratios (including soil C/N, C/P and N/P ratios), but positive relationships with soil total P and available P (Fig. 2).

### Specific microbial taxa drive changes in physiological traits and metabolic efficiency

There were significant shifts in taxonomic composition for bacterial community along the altitude gradient (PERMANOVA, *P* < 0.05; Fig. S2). The relative abundance of *Proteobacteria* and *Acidobacteria* increased, while that of *Cyanobacteria* declined with increasing altitude (Table S1). Other dominant bacterial phyla, including *Actinobacteria*, *Chloroflexi*, *Bacteroidetes*, *Gemmatimonadetes* and *Firmicutes*, were independent of altitude (*P* > 0.05; Table S1), although some of these phyla showed significant associations with soil abiotic factors (Fig. S3). The overall fungal community composition and the relative abundances of all the dominant fungal phyla, including *Ascomycota*, *Basidiomycota* and *Mortierellomycota*, showed no significant variations along the altitude gradient (Table S1; Fig. S2). Among these microbial phyla that varied with altitude, only *Proteobacteria* and *Cyanobacteria* had significant associations with microbial physiological traits and metabolic efficiency (Table S2).

**Fig. 3:**
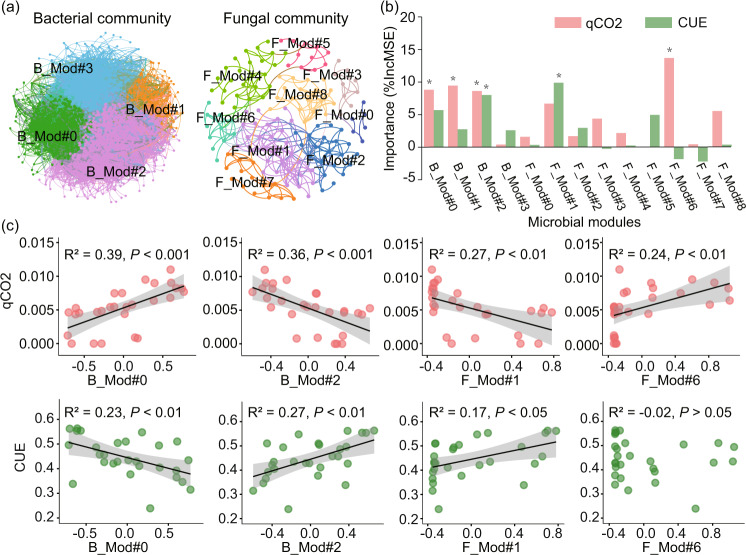
Major bacterial and fungal assemblies explaining microbial metabolic efficiency. **a** Network diagram with nodes (OTUs) colored by each of the major ecological modules (assemblies) within co-occurrence network of bacterial and fungal community, respectively; **b** key bacterial and fungal assemblies predicting metabolic quotient (qCO_2_) and carbon use efficiency (CUE) according to Random Forest analysis; **c** the relationships of key bacterial and fungal assemblies with metabolic efficiency. *P < 0.05; **P < 0.01.

In contrast, results of network analyses showed significant changes in the relative abundance of different microbial assemblies (clusters of microbial phylotypes that highly correlated with each other) along the altitude (Figs. 3 and S4). The soil bacterial and fungal phylotypes could be grouped into four (B_Mod#0-3) and eight (F_Mod#0-7) major microbial assemblies, respectively. Over these assemblies, the abundances of phylotypes belonging to B_Mod#0 and F_Mod#6 increased significantly with increasing altitude, while those belonging to B_Mod#2 and F_Mod#1 exhibited opposite patterns (Fig. S4). More importantly, these shifted bacterial and fungal assemblies with altitude were essential predictors of the variations in Vlength, qCO_2_ and CUE, even considering multiple edaphic properties (Figs. 3 and S5). Specifically, the relative abundance of B_Mod#0 was negatively related to Vlength and CUE, but positively to qCO_2_ and P-acquiring enzyme activity (Figs. 3 and S4). On the contrary, the relative abundance of B_Mod#2 showed totally reversed relationships with these potential resource acquisition strategies and metabolic efficiency. For fungal community, the relative abundance of F_Mod#1 showed generally positive relationships with Vlength and CUE, but negatively with qCO_2_ and P-acquiring enzyme activity. The proportion of F_Mod#6 correlated positively with qCO_2_ and enzyme activities involved in C, N and P acquirements (Figs. 3 and S4).

**Fig. 4:**
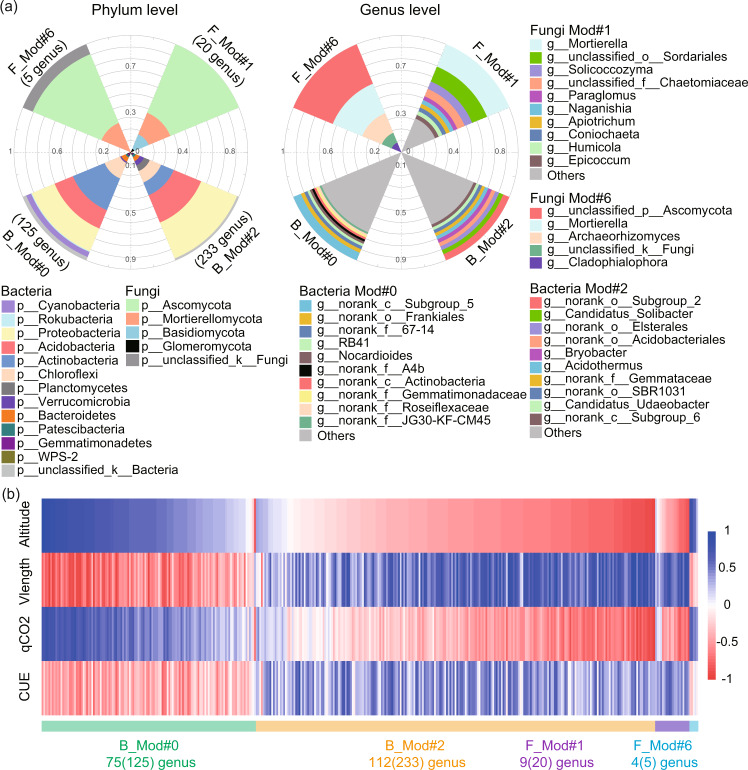
Microbial taxa in key bacterial and fungal assemblies drive changes in microbial trait-based strategies and microbial metabolic efficiency. **a** The proportions of dominant phyla and genus in key bacterial and fungal assemblies; **b** relationships of the relative abundances of different genus in each key assemblies with microbial physiological traits and metabolic efficiency. B_Mod#0 and B_Mod#2 were key bacterial assemblies, and F_Mod#1 and F_Mod#6 were key fungal assemblies. Vlength, the relative C:nutrient-acquiring traits. The number of genus that correlated significantly (*P* < 0.05) with altitude in each key assemblies were shown. The number in parentheses indicates the total number of genus in each key assemblies.

The proportion of most genera within B_Mod#0 and F_Mod#6 increased with increasing altitude, which also showed negative relationships with Vlength and CUE, but positive relationships with qCO_2_ (Fig. 4b). In contrast, the proportions of most genera within B_Mod#2 and F_Mod#1 declined as the altitude increased, and correlated positively with Vlength and CUE, but negatively with qCO_2_. The key assemblies for bacterial community were dominated by *Proteobacteria*, *Actinobacteria*, *Acidobacteria* and *Chloroflexi* (Fig. 4a). Most of the genera within B_Mod#0 belonged to the *Proteobacteria* (57 out of 125) and *Acidobacteria* (18 out of 125) (Table S2). For instance, genera *Bryobacter*, *GAS113*, *Edaphobacter*, *Roseiarcus*, and *Granulicella* all showed significantly positive associations with altitude and qCO_2_, but negatively with Vlength and CUE (Table S2). The majority of genera within B_Mod#2 belonged to the *Proteobacteria* (77 out of 233) and *Actinobacteria* (67 out of 233). In particular, genera such as *Sphingomonas*, *Nordella*, *Microlunatus*, *Rhizobacter* and *Lysobacter*, all showed negative relationships with altitude and qCO_2_, but positive correlations with Vlength and CUE. For fungal community, most of the phylotypes related to altitude were within *Ascomycota*, with *Coniochaeta*, *Neurospora*, *Epicoccum* and *Mortierella* etc. as essential genera predicting Vlength and metabolic efficiency (Table S2).

## Discussion

Our study established empirical associations between shifts in microbial community composition with different trait combinations and metabolic efficiency, accounting for internal controls over microbial metabolic efficiency and the associated ecosystem functions. We show that increasing C cost of maintenance (i.e., qCO_2_) and decreasing CUE was linked to increasing nutrient (particularly P) acquisition strategies along the altitude gradient. These results indicate that in soils with lower substrate quality (higher C:N and C:P ratios), resource limitations drive microbial communities to invest more energy into resource acquisition strategies that trade-off against growth yield at higher altitude. Such a trade-off of physiological traits could be related to shifts in microbial taxa with distinct trait-based strategies. In particular, the shifted bacterial and fungal assemblies with altitude were also essential predictors of microbial resource acquisition strategies and metabolic efficiency. Based on the framework of trait-based ecology, our data provide evidence for the linkages between shifts in microbial community composition and community-level metabolic efficiency along a broad environmental gradient. These findings illustrate that the intrinsic properties of microbial community play crucial roles in mediating the efficiency of microbial processes such as CUE, highlighting that the incorporation of intrinsic microbial properties into models is important to better predict biogeochemical cycles and their feedbacks to climate changes.

Consistent with our first hypothesis, the metabolic efficiency of microbial community was low (higher qCO_2_ and lower CUE) at higher altitude, partly due to the higher energy investments in nutrient acquisition strategies (Fig. 1; Table S1). At higher altitude with lower substrate quality (higher soil C:N and C:P ratios), the lower Vlength and higher specific P-acquiring enzyme activities indicate that more energy are allocated to acquire the most limited nutrients (N and P) under unbalanced resource stoichiometry [15]. The production of extracellular enzymes to acquire nutrients from complex biomolecules is energetically expensive, and thus may contribute to higher C cost of maintenance and lower CUE at higher altitude [3]. In support of this, we found that the specific P- acquiring enzyme activities correlated negatively with CUE (Fig. 2), indicating that increasing microbial trait strategies of resource (P) acquisition through enzymes reduced metabolic efficiency at higher altitude. Similar relative nutrient (N and P) constraints to microbial metabolism were also predicted in high-latitude grasslands with low temperature using a new enzymatic stoichiometric model [49]. Our results provide evidence for a clear trade-off between community-level resource acquisition potential and growth yield based on empirical relationships along a highly heterogeneous environmental gradient.

Our study also presents that a large amount of variation in metabolic efficiency was explained by altitude, which correlated highly (*R*^2^ = 0.989) with temperature in these harsh alpine environments [25, 26]. Therefore, it is plausible that growth traits also trade-off with cold-stress tolerance strategies along the altitude gradient in the highest plateau on Earth. Many previous studies suggested that low temperature could inhibit the growth and metabolic activity of microorganisms [50, 51]. Traits involved in cold adaptions such as biofilm formation and membrane modifications were favored under cold environments [52, 53], which may contribute to the lower metabolic efficiency under cold conditions. As such, the harsh environments (low substrate quality and temperature) may induce trade-offs among multiple strategies including growth yield, resource acquisition and stress tolerance. Future works on physiological adaptions of microbial communities to cold-stress tolerance and their effects on microbial metabolic efficiency will consolidate our findings.

Additionally, microbial taxonomic variations were highlighted as essential predictors of metabolic efficiency according to our results of Random Forest analysis (Fig. 2). This supports previous studies that community-level variations in physiological traits mostly reflect the legacy effects of long-term environmental changes on microbial community composition [12]. In this study, soil samples were taken in the last month of the growing season, when soil availability is high and the immediate freeze-thawing effects can be disregarded. As such, our study could provide a snapshot of shifts in microbial community composition and trait strategies across a low temperature gradient with distinct changes in abiotic factors (including soil pH, substrate quantity and quality). Soils at higher altitude are likely to host microbial taxa that adapted to low substrate quality and temperature [54, 55]. In support of this, we observed increased abundance of *Proteobacteria* with increasing altitude, which is always dominant in cold and oligotrophic conditions [52, 56, 57]. Furthermore, the relative abundance of *Proteobacteria* associated positively with the potential functions involved in nutrient acquisition (i.e., lower Vlength) and qCO_2_ (Table S1), indicating that environmental selection of specific taxa contribute to community-level changes in physiological traits and metabolic efficiency [12]. However, except for *Proteobacteria*, most dominant bacterial and fungal phyla were independent of altitude and acted as weak predictors of changes in physiological traits and metabolic efficiency. These results indicate that microbial classification based on higher phylogenetic levels (such as at the order or phylum levels) have limitations in explaining community-level physiological strategies, because taxonomically related strains can have divergent features, while dissimilar strains may exhibit identical traits [20, 21].

Microbial community could also be grouped into assemblies with particular trait combinations based on different co-occurrence or association patterns, offering new insights into complex microbial community structure and soil functioning [10, 22]. Microbial assemblies within co-occurrence network are significant predictors of microbial physiological traits, even considering other key environmental attributes (Figs. 3 and S5). In particular, the relative abundance of B_Mod#0 increased significantly with increasing altitude (concurrent declining temperature) and soil C:N and C:P ratios (Fig. S4), suggesting that these taxa adapted to low temperature and substrate quality at higher altitude. Interestingly, these adapted taxa within B_Mod#0 also showed significant associations with community-level microbial nutrient acquisition traits, qCO_2_ and CUE, further indicating that environmental selection of specific taxa drives changes in physiological traits and the metabolic efficiency of microbial community. Most of genera within B_Mod#0 were from *Alphaproteobacteria* and *Acidobacteria*, which were mostly *K*-strategists (slow-growing equilibrium species) and also the main sources of assumed cold-adaption genes such as those encoding OstA (trehalose phosphate synthase), OstB (trehalose phosphatase) and Fatty acid desaturases etc. [52]. In contrast, B_Mod#2 may be mainly composed of microbial groups with low maintenance efforts and weak adaption to low temperature and nutrient limitation, as illustrated by its negative relationships with altitude, soil C:nutrient ratios and the associated physiological traits. Therefore, the shift of bacterial assemblies from B_Mod#2 to B_Mod#0 with different trait strategies appears to be an important control on the increased C cost via maintenance and declined CUE with increasing altitude.

**Fig. 5:**
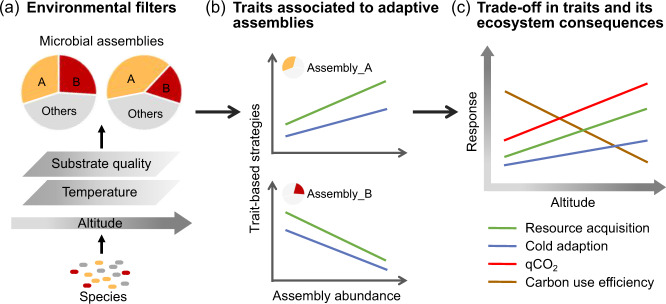
A conceptual paradigm incorporating trait-based framework to predict metabolic efficiency under environmental changes. **a** Shift in microbial community composition under environmental changes; **b** changes in the proportion of microbial assemblies with distinct adaptive traits along the altitude gradient; **c** trade-off in different microbial physiological traits and its consequences on metabolic efficiency at the community level.

Similarly, shifts of fungal assemblies could also contribute to the variation in the metabolic efficiency along the altitude gradient. Microbial taxa within F_Mod#6 may have slow-growing rates, as these taxa generally showed positive associations with qCO_2_, but negative relationships with CUE (Fig. 4b; Table S2). For instance, genus *Mortierella*, as the keystone genus for F_Mod#6, were reported as essential psychrophiles and could produce high abundant trehalose and fatty acids to adapt to cold stress [51, 58]. In contrast, the keystone genus consisted of F_Mod#1, such as genera *Neurospora* was reported to have fast-growing lifestyles [59] and distributed mainly in warm regions like tropical and subtropical areas [60]. Overall, it is plausible that microbial taxa belonged to B_Mod#0 and F_Mod#6 adapted to low temperature and resource quality conditions at higher altitude, but at the expense of more C allocated to maintenance and lead to less efficient microbial community at higher altitude. Our findings link complex microbial community structure to functions under the framework of trait-based ecology, and highlight the importance of shifts in microbial functional groups with different traits for the metabolic efficiency of microbial community (Fig. 5).

While our study provides the empirical evidence for the roles of intrinsic properties of microbial community in regulating metabolic efficiency, these effects of biotic properties may be conflated with those of abiotic factors along the altitude gradient. For instance, declined soil pH significantly enhanced qCO_2_ and reduced microbial CUE, consistent with a recent study showing that acidic soils had very slow microbial growth rates and low CUE due to physiological constraints [61]. Moreover, higher qCO_2_ could also be interpreted as more soil C (substrate) being available for microbial respiration at higher altitude [4]. However, it should be noted that biotic properties, particularly microbial resource acquisition traits and essential microbial assemblies, were important predictors of community-level metabolic efficiency even after considering these key abiotic factors. Therefore, our findings highlight that shifts in microbial assemblies with distinct life-history strategies should be incorporated into current trait-based models to better predict microbial community responses and the associated ecosystem functions under environmental changes (Fig. 5). Overall, microbial adaptions due to shifts in physiological traits and community composition lead to less efficient microbial community and lower energy investment into microbial growth and biomass production, which may further reduce the rates of essential ecosystem processes including soil organic matter decomposition and nutrient turnover at higher altitude.

## Conclusions

To conclude, our results demonstrate that shifts in microbial assemblies with distinct physiological traits drive changes in community-level metabolic efficiency along the altitude gradient. At higher altitude, resource limitations drive microbial communities to invest more energy into resource acquisition strategies that trade off against growth yield, leading to higher metabolic quotient and lower CUE. These different microbial physiological traits could reflect the shifts in microbial functional groups with divergent strategies for coping with environmental constraints. We suggest that microbial taxa can adapt to nutrient limited and cold conditions at higher altitude, but at the expense of more maintenance efforts and lower metabolic efficiency. Our results highlight the shifts in trait-based strategies and microbial functional groups as essential factors regulating the metabolic efficiency of microbial community. This work could have important implications for C cycle in a changing world, and thus improve the predictive understanding of soil C responses to future climate change.

## Supplementary information

Supporting Information

Table S2
