# Supplementary material for: Soil microbial trait-based strategies drive metabolic efficiency along an altitude gradient
Source: ISME Commun. 2021 Dec 3;1:71. doi: 10.1038/s43705-021-00076-2 (PMC9723748; doi:10.1038/s43705-021-00076-2)
Supplement: Supplementary file 1 — Supporting Information [file 43705_2021_76_MOESM1_ESM.docx]

**Table S1.** Correlation coefficients (Spearman’s rank correlation coefficient) between the relative abundances of dominant bacterial and fungal phyla and soil microbial trait-based strategies. *P*-values below 0.05 are in bold.

|  | Dominant Phyla | Altitude | Vlength | qCO_2_ | CUE |
| --- | --- | --- | --- | --- | --- |
| Bacteria | Proteobacteria | **0.68** | **-0.59** | **0.69** | -0.35 |
|  | Actinobacteria | -0.44 | 0.35 | -0.40 | 0.22 |
|  | Acidobacteria | **0.56** | -0.43 | 0.42 | -0.23 |
|  | Chloroflexi | -0.34 | 0.44 | -0.21 | 0.32 |
|  | Bacteroidetes | -0.17 | 0.21 | 0.04 | 0.34 |
|  | Gemmatimonadetes | -0.39 | **0.50** | -0.34 | 0.29 |
|  | Cyanobacteria | **-0.66** | 0.38 | **-0.58** | -0.01 |
|  | Verrucomicrobia | 0.23 | -0.26 | 0.23 | -0.20 |
|  | Patescibacteria | -0.30 | 0.06 | -0.14 | 0.11 |
|  | Planctomycetes | 0.36 | -0.40 | 0.27 | -0.34 |
|  | Firmicutes | -0.22 | 0.33 | -0.18 | 0.34 |
| Fungi | Ascomycota | -0.12 | 0.31 | -0.16 | 0.18 |
|  | Basidiomycota | 0.32 | -0.40 | 0.20 | -0.34 |
|  | Mortierellomycota | -0.19 | 0.22 | -0.04 | 0.42 |
|  | Glomeromycota | 0.07 | 0.09 | -0.15 | 0.12 |
|  | Chytridiomycota | -0.53 | 0.55 | **-0.59** | 0.35 |
|  | Entorrhizomycota | 0.04 | 0.07 | -0.09 | 0.18 |
|  | Olpidiomycota | 0.13 | 0.39 | 0.22 | 0.50 |
|  | Rozellomycota | 0.18 | -0.04 | 0.21 | 0.04 |
|  | Blastocladiomycota | -0.23 | 0.23 | -0.29 | 0.19 |

Note: Vlength, the relative C vs. nutrient (N and P)-acquiring strategies, lower Vlength indicated relative higher nutrient acquisition strategies (relative to C); CUE, carbon use efficiency.

**Table S2.** Relationships of the relative abundances of genus belonged to key bacterial and fungal assemblies with metabolic efficiency and microbial physiological traits.

**
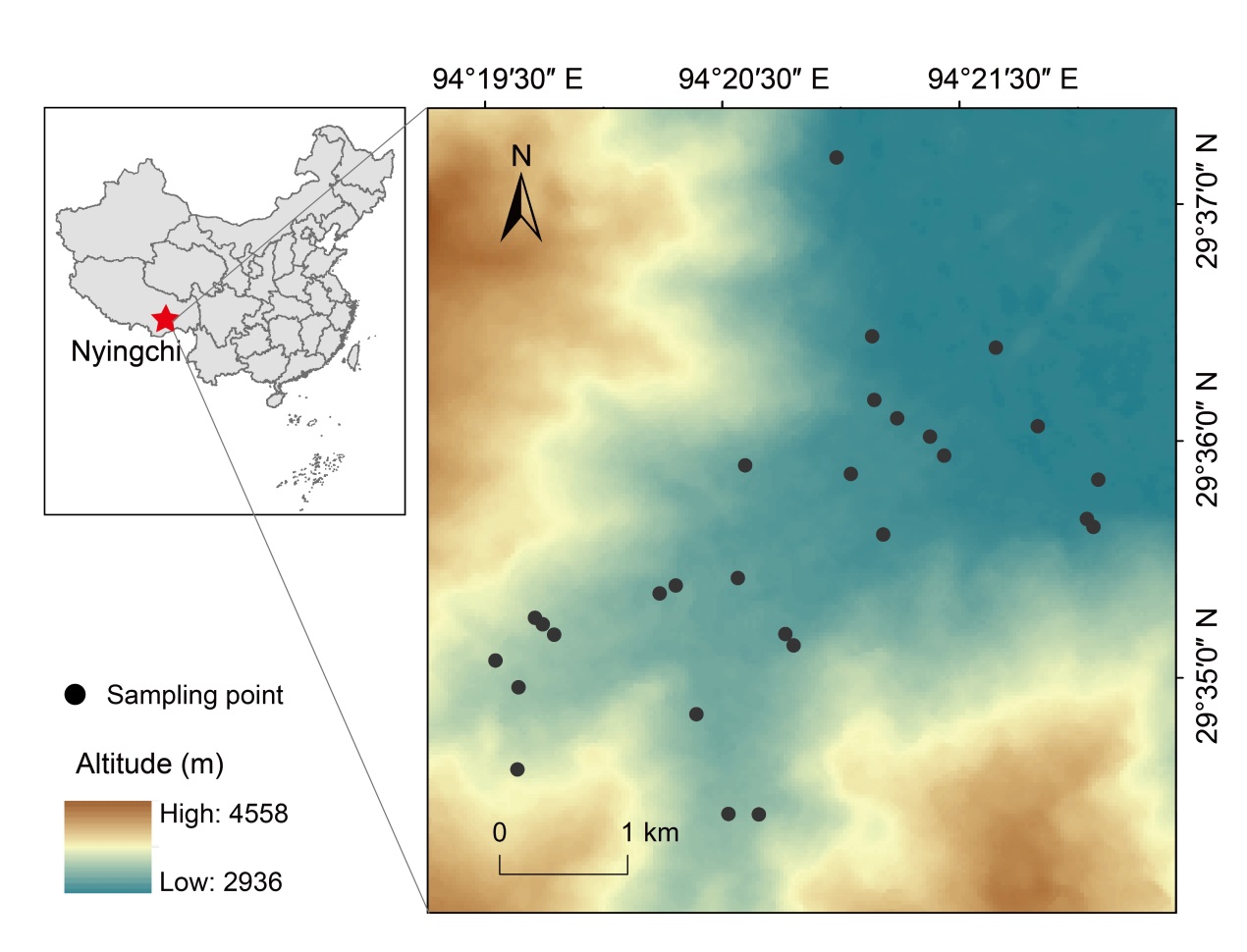
Figure S1.** Distribution of soil sampling sites along the altitude gradient on the Tibetan Plateau.

**
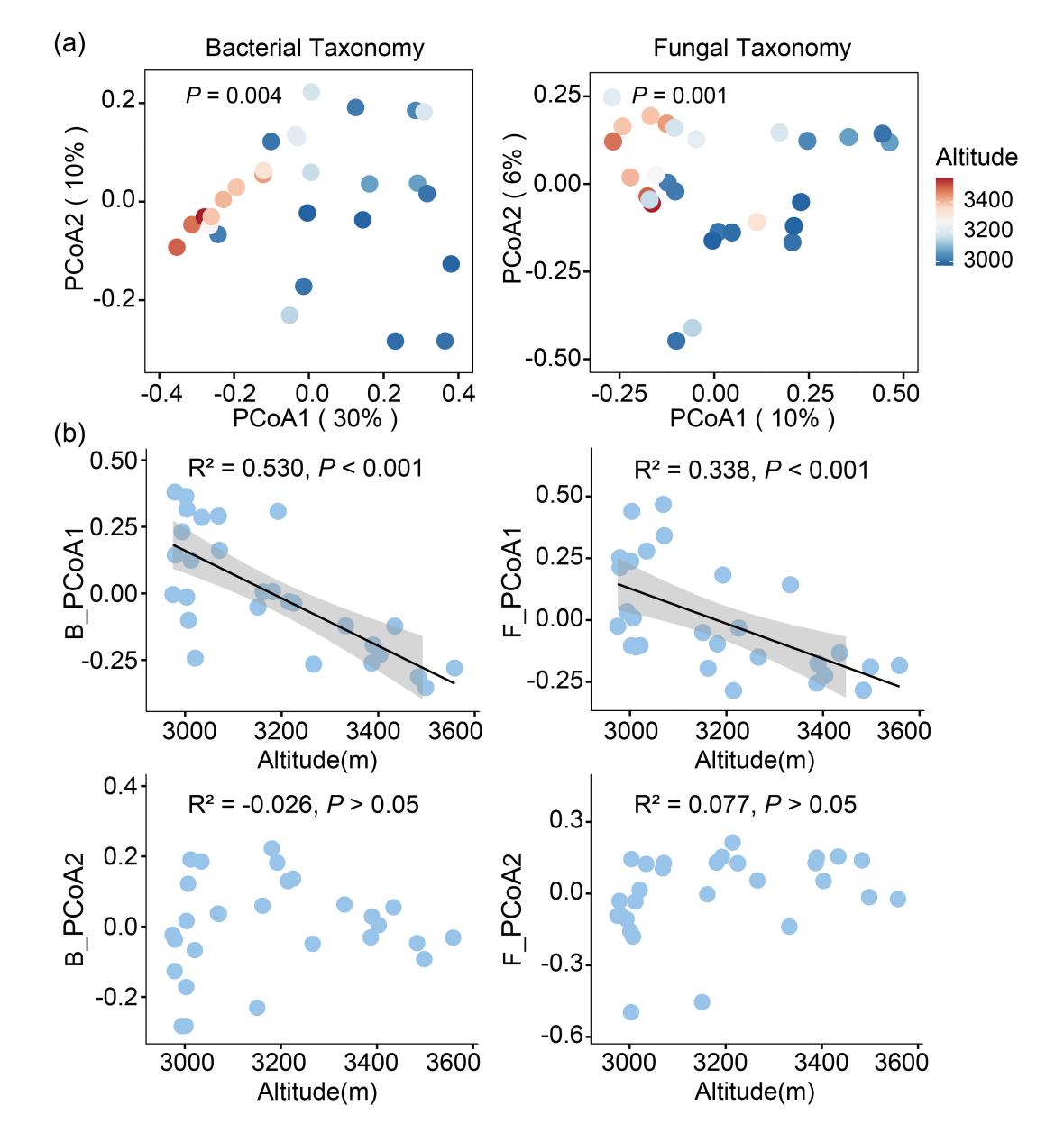
**

**Figure S2.** Taxonomic composition for bacterial and fungal communities along the altitude gradient. (a) Principal coordinate analysis (PCoA) of taxonomic composition for bacterial and fungal communities; (b) patterns of the two dimensional PCoA ordination of taxonomic composition for bacterial and fungal community, respectively. Statistical differences in the taxonomic community composition were tested using PERMANOVA (*P* < 0.05). H, high altitude (altitude ≥ 3300 m); M, middle altitude (3100 ≤ altitude < 3300 m); L, low altitude (2900 ≤ altitude < 3100 m).

**
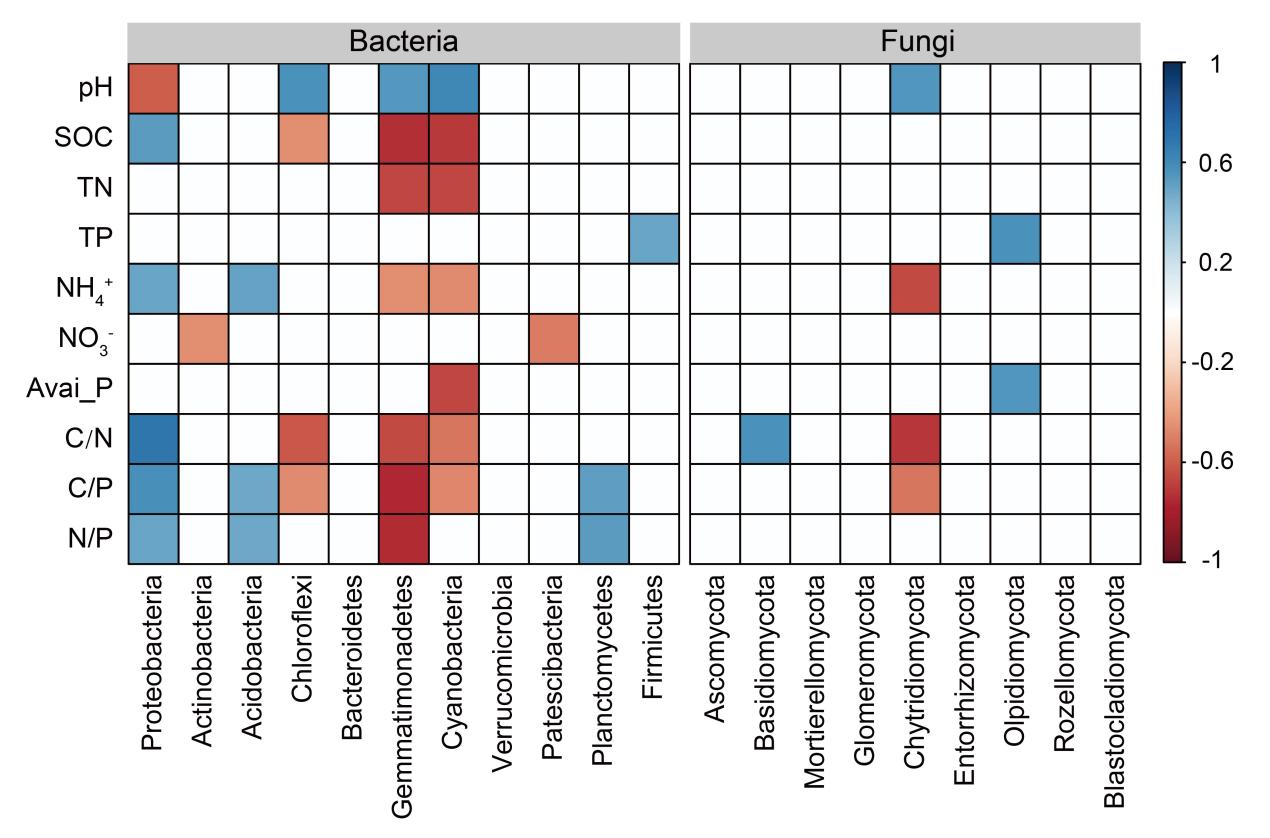
**

**Figure S3.** Relationships between the relative abundances of the dominant bacterial and fungal phyla and environmental factors (altitude, soil substrate quantity and quality). SOC, soil organic C; TN, total N; TP, total P; Avai_P, available P.


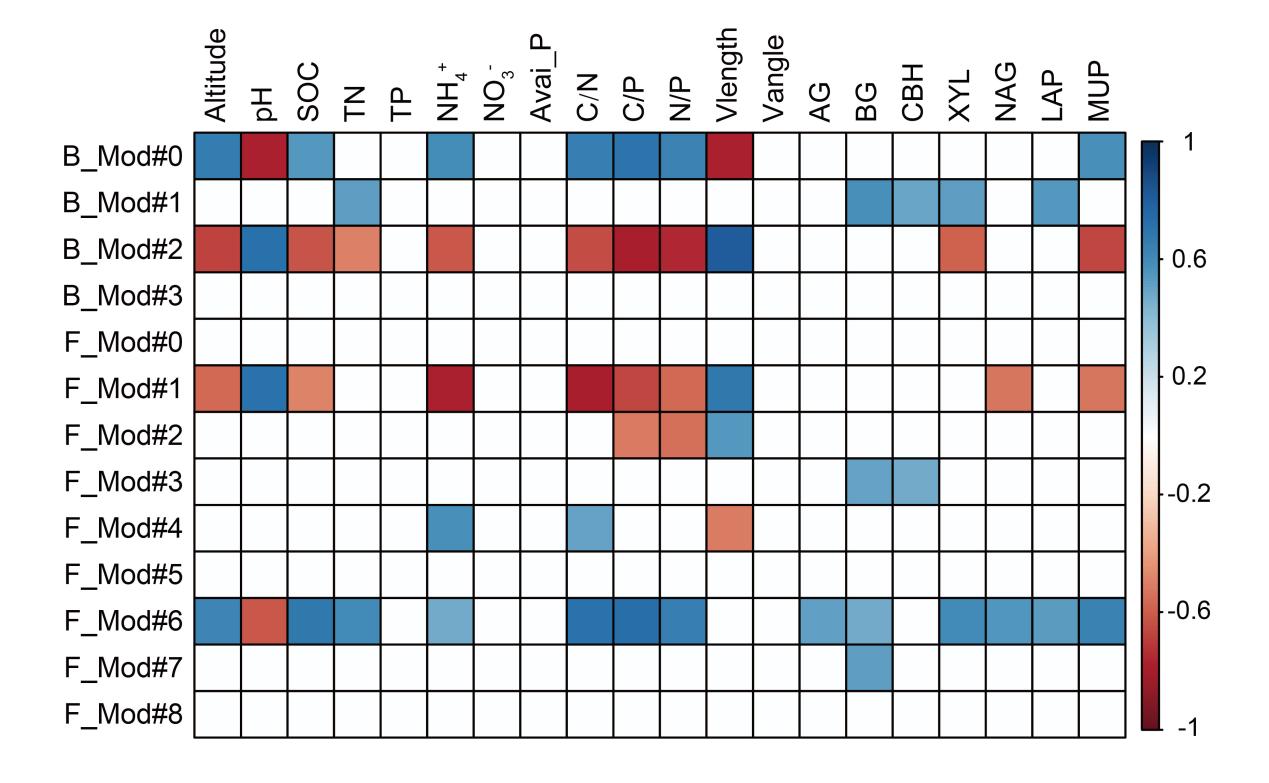


**Figure S4.** Relationships between the relative abundances of major bacterial and fungal assemblies and environmental factors (altitude, soil substrate quantity and quality). SOC, soil organic C; TN, total N; TP, total P; Avai_P, available P.

**
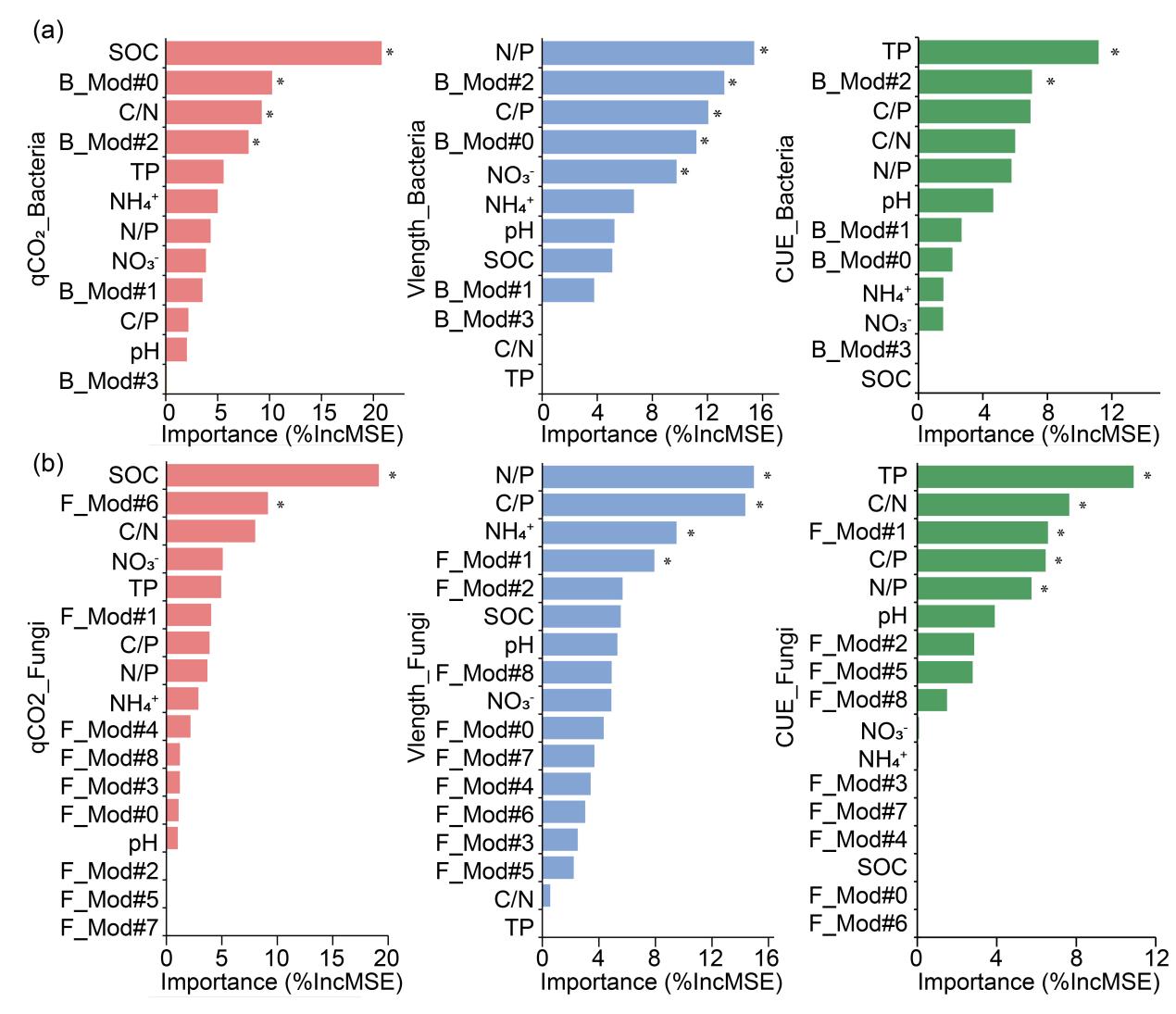
**

**Figure S5.** Essential predictors for metabolic efficiency and microbial physiological trait-based strategies. %IncMSE, the percentage increases in the mean squared error (%IncMSE) based on Random Forest analysis by incorporating both microbial assemblies and other key environmental factors. * *P* < 0.05.
